# Some limit properties for a hidden inhomogeneous Markov chain

**DOI:** 10.1186/s13660-018-1884-7

**Published:** 2018-10-25

**Authors:** Yun Dong, Fang-qing Ding, Qi-feng Yao

**Affiliations:** 1 School of Mathematics, Maanshan Teachers’ College, Maanshan, China; 2 Department of Mathematics and Physics, HeFei University, Anhui, P.R. China; 3 School of Mathematics & Physics, AnHui University of Technology, Ma’anshan, China

**Keywords:** 60F15, 94A17, Inhomogeneous hidden Markov chain, Delayed sum, Law of large numbers

## Abstract

This paper presents a general strong limit theorem for delayed sum of functions of random variables for a hidden time inhomogeneous Markov chain (HTIMC), and as corollaries, some strong laws of large numbers for HTIMC are established thereby.

## Introduction

Hidden Markov chain is an important branch of Markov chain theory. A classical hidden Markov model was first introduced by Baum and Petrie [1]. It provides a flexible model that is very useful in different areas of applied probability and statistics. Examples are found in machine recognition, like speech and optical character recognition, and bioinformatics. The power of these models is that they can be very efficiently implemented and simulated. In recent years, many new theories were introduced into hidden time inhomogeneous Markov chain (HTIMC) theory. G.Q. Yang et al. [2] gave a law of large numbers for countable hidden time inhomogeneous Markov models. In addition, delayed sums of random variables were first discussed by Zygmund [3]. Gut and Stradtmüller [4] studied the strong law of large numbers for delayed sums of random fields. Wang and Yang [5] studied the generalized entropy ergodic theorem with a.e. and $\mathcal{L}_{1} $ convergence for time inhomogeneous Markov chains. Wang [6, 7] discussed the limit theorems of delayed sums for row-wise conditionally independent stochastic arrays and a class of asymptotic properties of moving averages for Markov chains in Markovian environments.

In the classical studies there are two simplest models for predicting: the mean model and the random walk model [8]. These two models use all the historical information. But we often encounter time series that appear to be “locally stationary”, so we can take an average of what has happened in some window of the recent past. Based on this idea and the above researches, the main focus of this paper is to obtain a general strong limit theorem of delayed sums of functions of random variables for an HTIMC, and as corollaries, some strong laws of large numbers for HTIMC are established thereby.

The remainder of this paper is organized as follows: Sect. 2 gives a brief description of the HTIMC and related lemmas. Section 3 presents the main results and the proofs.

## Preliminaries

In this section we list some fundamental definitions and related results that are needed in the next section.

Let $( \Omega , \mathcal{F} , \mathbb{P} )$ be the underling probability space and $\zeta = ( \xi , \eta )$ a random vector, where $\xi = ( \xi _{0} , \xi _{1} ,\ldots )$ and $\eta = ( \eta _{0} , \eta _{1} ,\ldots )$ are two different stochastic processes, *η* is hidden (*η* takes values in set $\mathcal{Y} = \{ \omega _{0} , \omega _{1} ,\ldots, \omega _{b} \}$) and *ξ* is observable (*ξ* takes values in set $\mathcal{X} = \{ \theta _{0} , \theta _{1} ,\ldots, \theta _{d} \}$).

We first recall the definition of a hidden time inhomogeneous Markov chain (HTIMC) $\zeta = ( \xi , \eta ) = \{ \xi_{n} , \eta_{n} \} _{n=0}^{\infty } $ with hidden chain $\{ \eta_{n} \}_{n=0}^{\infty }$ and observable process $\{ \xi_{n} \}_{n=0}^{\infty }$.

### Definition 1

The process $\zeta = ( \xi , \eta )$ is called an HTIMC if it follows the following form and conditions:

1. Suppose that a given time inhomogeneous Markov chain takes values in state space $\mathcal{Y}$, its starting distribution is
  2.1 $$ \bigl(q(\omega _{0} ), q(\omega _{1} ); \ldots ; q( \omega_{b})\bigr),\quad q(\omega_{i}) > 0, \omega_{i}\in \mathcal{Y}, $$
  and transition matrices are
  2.2 $$ \mathcal{Q} _{k} = \bigl( q _{k} ( \omega_{j } \mid \omega_{i} )\bigr),\quad q _{k} ( \omega_{j } \mid \omega_{i} ) > 0, \omega_{i} , \omega_{j} \in \mathcal{Y} , k \geq 1, $$
  where
  $$q _{k} ( \omega_{j} \mid \omega_{i} ) = \mathbb{P} ( \eta_{k} = \omega_{j} \mid \eta _{ k-1 } = \omega_{i} ), \quad k \geq 1. $$
2. For any positive integer *n*,
  2.3 $$ \mathbb{P} ( \xi _{0} = x _{0} ,\ldots, \xi_{n} = x _{n} \mid \eta ) = \prod_{k =0}^{n } \mathbb{P} ( \xi_{k} = x_{k} \mid \eta_{k} ) \quad \text{a.s.} $$

Some necessary and sufficient conditions for (2.3) have been given by G.Q. Yang et al. [2].

- (2.3) holds if, for any *n*,
  2.4 $$ \mathbb{P} ( \xi _{0} = x _{0} ,\ldots, \xi_{n} = x _{n} \mid \eta _{0} =y _{0} ,\ldots, \eta_{n} =y _{n} ) = \prod_{k =0}^{n } \mathbb{P} ( \xi_{k} = x_{k} \mid \eta_{k} = y_{k} ) $$
  holds.
- $\zeta = ( \xi , \eta )$ is a hidden time inhomogeneous Markov chain if and only if $\forall n \geq 0$,
  2.5 $$ p(x_{0} , y_{0},\ldots, x_{n}, y_{n}) = q(y_{0} )\prod_{k=1}^{n} q_{k} ( y_{k} \mid y_{k-1} ) \prod _{k=0}^{n} p_{k} ( x_{k} \mid y_{k} ), \quad n\geq 1. $$
- $\zeta = ( \xi , \eta )$ is a hidden time inhomogeneous Markov chain if and only if $\forall n \geq 0$,
  2.6 $$\begin{aligned}& \begin{aligned}[b] &\mathbb{P} ( \eta_{n} = y _{n} \mid \xi _{0} = x _{0} ,\ldots, \xi _{ n-1 } = x _{ n-1 }, \eta _{0} = y _{0} ,\ldots, \eta _{ n-1 } = y _{ n-1 }) \\ &\quad = \mathbb{P} ( \eta_{n} = y _{n} \mid \eta _{ n-1 } = y _{ n-1 }), \end{aligned} \end{aligned}$$
  2.7 $$\begin{aligned}& \begin{aligned}[b] & \mathbb{P} ( \xi_{n} = x _{n} \mid \xi _{0} = x _{0} ,\ldots, \xi_{n} = x _{n} , \eta _{0} = y _{0} ,\ldots, \eta _{ n-1 } = y _{ n-1 }) \\&\quad = \mathbb{P} ( \xi_{n} = x _{n} \mid \eta_{n} = y _{n} ). \end{aligned} \end{aligned}$$

Let $\{ a_{n}, b_{n} \} $ be two sequences of nonnegative integers with $b _{n}$ converging to infinity as $n \rightarrow \infty $. Let $\mathcal{S}_{a_{n}, b_{n}} ( \theta_{i} , \omega_{j} )$, $\mathcal{W}_{a_{n}, b_{n}} ( \omega_{i } )$, $\mathcal{T}_{a _{n}, b_{n}} ( \theta_{i} )$, $\theta_{i} \in \mathcal{X}$, $\omega _{j} \in \mathcal{Y}$ be the number of ordered couples $( \theta_{i} , \omega_{j} )$ in $( \xi_{a_{n} +1} , \eta_{a_{n} +1} ),( \xi_{a_{n} +2} , \eta_{a_{n} +2} ),\ldots,( \xi_{a_{n} + b_{n}} , \eta_{a_{n} + b _{n}} )$, with $\omega_{i}$ among $\eta_{a_{n} +1} , \eta_{a_{n} +2} ,\ldots, \eta_{a_{n} + b_{n}} $ and $\theta_{i}$ among $\xi_{a_{n} +1} , \xi_{a_{n} +2},\ldots, \xi_{a_{n} + b_{n}} $, respectively.

It is easy to verify that

2.8 $$\begin{aligned}& \mathcal{S}_{a_{n}, b_{n}}(\theta_{i}, \omega_{j}) = \sum _{k= a_{n} +1} ^{a_{n} + b_{n}} 1_{ \{ \theta_{i} \} } ( \xi_{k} ) 1_{ \{ \omega_{j} \} } ( \eta_{k} ) , \end{aligned}$$

2.9 $$\begin{aligned}& \mathcal{W}_{a_{n}, b_{n}} ( \omega_{i} )=\sum _{k= a_{n} +1}^{a_{n} + b_{n}} 1_{ \{ \omega_{i} \} } ( \eta_{k} ) , \end{aligned}$$

and

2.10 $$ \mathcal{T}_{a_{n}, b_{n}}(\theta_{i})=\sum _{k= a_{n} +1}^{a_{n} + b _{n}} 1_{ \{ \theta_{i} \} } ( \xi_{k} ) , $$

where $1_{A}(\cdot )$ denotes the indicator function of set *A*.

### Lemma 1

*Let* $\zeta = ( \xi , \eta ) = \{ ( \xi _{k}, \eta_{k} ) \} _{k =0}^{\infty }$ *be an HTIMC which takes values in* $\mathcal{X} \times \mathcal{Y}$, *let* $\{ f_{k} (x,y) \} _{k =0}^{\infty }$ *be a sequence of functions on* $\mathcal{X} \times \mathcal{Y}$, *let* $\mathcal{F} _{m,n} = \sigma \{ ( \xi_{m} , \eta_{m} ,\ldots, \xi_{n} , \eta _{n} ),0 \leq m \leq n \in Z _{+} \}$, *and let* $\{ a _{n} , b _{n} \}$ *be a sequence of pairs of positive integers with* $\sum_{n =1}^{\infty } \exp [ - \varepsilon b_{n} ] < \infty $, *where* $\varepsilon > 0$ *is arbitrary*. *Define*

2.11 $$ \begin{aligned}[b] &A(\alpha ) =\Biggl\{ \omega : \mathop{\lim \sup}_{n\rightarrow \infty } \frac{1}{b _{n}} \sum_{k= a_{n} +1}^{a_{n} + b_{n}} \mathbb{E} \bigl[ f_{k}^{2} ( \xi_{k}, \eta_{k} ) e^{\alpha \vert f_{k} ( \xi _{k}, \eta_{k} ) \vert } \mid \mathcal{F}_{a_{n},k-1} \bigr] =M ( \alpha ,\omega ) < \infty \Biggr\} \\ &\quad (\alpha >0). \end{aligned} $$

*Then*

2.12 $$ \lim_{n\rightarrow \infty } \frac{1}{b_{n}} \sum _{k= a_{n} +1}^{a_{n} + b_{n}} \bigl\{ f_{k} ( \xi_{k}, \eta_{k} ) - \mathbb{E} \bigl[ f_{k} ( \xi_{k}, \eta_{k} ) \mid \mathcal{F}_{a_{n},k-1} \bigr] \bigr\} =0 \quad \textit{a.s. }\omega \in A(\alpha ). $$

### Proof

Let *λ* be a real number. We first define

2.13 $$ t_{a_{n}, b_{n}} (\lambda ,\omega )= \frac{e^{\lambda \sum_{k= a_{n} +1} ^{a_{n} + b_{n}} f_{k} ( \xi_{k}, \eta_{k} ) }}{ \prod_{k= a_{n} +1}^{a_{n} + b_{n}} \mathbb{E} [ e^{\lambda f _{k} ( \xi_{k} ,\eta_{k} )} \mid \mathcal{F}_{a_{n},k-1} ] }. $$

Note that

$$ t_{a_{n}, b_{n}} (\lambda ,\omega )= t_{a_{n}, b_{n-1}} (\lambda , \omega ) \frac{e^{\lambda f_{a_{n} + b_{n}} ( \xi_{a n + b n}, \eta_{a n + b n} )}}{\mathbb{E} [ e^{\lambda f_{a_{n} + b_{n}} ( \xi_{a n + b n}, \eta_{a n + b n} )} \mid \mathcal{F}_{a_{n}, a_{n} + b_{n} -1} ] } $$

and

$$\mathbb{E} \bigl[ t_{a_{n}, b_{n}} (\lambda ,\omega ) \bigr] = \mathbb{E} \bigl\{ \mathbb{E} \bigl[ t_{a_{n}, b_{n}} ( \lambda ,\omega ) \bigr]\mid \mathcal{F}_{a_{n}, a_{n} + b_{n} -1} \bigr\} . $$

Hence, we have

$$\mathbb{E} \bigl[ t_{a_{n}, b_{n}} (\lambda ,\omega ) \mid \mathcal{F}_{a_{n}, a_{n} + b_{n} -1} \bigr] = t_{a_{n}, b_{n-1}} (\lambda ,\omega ) \quad \text{a.s.} $$

It is easy to show that $\mathbb{E} [ t_{a_{n}, b_{n} ( \lambda ,\omega ) } ] = 1$; $\forall n \geq 1$. This and the Markov inequality imply that, for every $\varepsilon > 0$,

$$ \mathbb{P}\biggl[ \frac{1}{b_{n}} \log t_{a_{n}, b_{n}} (\lambda , \omega ) \geq \varepsilon \biggr] = \mathbb{P} \bigl[ t_{a_{n}, b_{n}} (\lambda ,\omega ) \geq \exp ( n\varepsilon ) \bigr] \leq 1 \cdot \exp (- \varepsilon b_{n} ). $$

Hence

$$ \sum_{n=1}^{\infty } \mathbb{P} \biggl[ \frac{1}{b_{n}} \log t_{a_{n}, b _{n}} (\lambda ,\omega ) \geq \varepsilon \biggr]\leq \sum_{n=1}^{\infty } \exp (-\varepsilon b_{n} )< \infty , $$

which, by the first Borel–Cantelli Lemma, allows us to conclude that $\lim\sup_{n} \frac{1}{ b_{n}} \log t_{a_{n}, b_{n}} (s, \omega ) < \varepsilon $ a.s., since *ε* is arbitrary, thus

2.14 $$ \mathop{\lim\sup}_{n\rightarrow \infty } \frac{1}{b_{n}} \log t_{a_{n}, b_{n}} ( \lambda ,\omega )\leq 0 \quad \text{a.s.} $$

follows since $\frac{1}{b_{n}} \log n^{2} = \frac{2 \log n}{b_{n}} \rightarrow 0$ ($n\rightarrow \infty $). We have by Eqs. (2.13) and (2.14) that

2.15 $$ \mathop{\lim\sup}_{n\rightarrow \infty } \frac{1}{b_{n}} \Biggl\{ \lambda \sum _{k= a_{n} +1}^{a_{n} + b_{n}} f_{k} ( \xi_{k} ,\eta _{k} ) - \sum_{k= a_{n} +1}^{a_{n} + b_{n}} \log \mathbb{E} \bigl[ e^{\lambda f_{k} ( \xi_{k} , \eta_{k} ) } \mid \mathcal{F}_{a_{n},k-1} \bigr] \Biggr\} \leq 0 \quad \text{a.s.} $$

Taking $0 < \lambda \leq \alpha $, and dividing both sides of Eq. (2.15) by *λ*, we get

2.16 $$ \mathop{\lim\sup}_{n\rightarrow \infty } \frac{1}{b_{n}} \Biggl\{ \sum _{k= a_{n} +1}^{a_{n} + b_{n}} f_{k} ( \xi_{k} ,\eta _{k} ) - \sum_{k= a_{n} +1}^{a_{n} + b_{n}} \frac{\log \mathbb{E} [ e^{\lambda f_{k} ( \xi_{k} , \eta_{k} ) } \mid \mathcal{F}_{a_{n},k-1} ]}{\lambda } \Biggr\} \leq 0 \quad \text{a.s.} $$

We have by Eq. (2.16) and inequalities $\log x \leq x-1$ ($x > 0$), $0 \leq e^{x} -1-x\leq \frac{1}{2} x^{2} e^{ \vert x \vert } $ that

2.17 $$\begin{aligned}& \mathop{\lim\sup}_{n\rightarrow \infty } \frac{1}{b_{n}} \sum _{k= a_{n} +1}^{a_{n} + b_{n}} \bigl\{ f_{k} ( \xi_{k} ,\eta_{k} ) - \mathbb{E} \bigl[ f_{k} ( \xi_{k}, \eta_{k} ) \mid \mathcal{F}_{a_{n},k-1} \bigr] \bigr\} \\ & \quad \leq \mathop{\lim\sup}_{n\rightarrow \infty } \frac{1}{b_{n}} \sum _{k= a_{n} +1}^{a_{n} + b_{n}} \biggl\{ \frac{\log \mathbb{E} [ e ^{\lambda f_{k} ( \xi_{k} , \eta_{k} ) } \mid \mathcal{F} _{a_{n},k-1} ]}{\lambda } - \mathbb{E} \bigl[ f_{k} ( \xi_{k}, \eta _{k} ) \mid \mathcal{F}_{a_{n},k-1} \bigr]\biggr\} \\ & \quad \leq \mathop{\lim\sup}_{n\rightarrow \infty } \frac{1}{b_{n}} \sum _{k= a_{n} +1}^{a_{n} + b_{n}} \biggl\{ \frac{\mathbb{E} [ e^{ \lambda f_{k} ( \xi_{k} , \eta_{k} ) } \mid \mathcal{F} _{a_{n},k-1} ] - 1}{\lambda } - \mathbb{E} \bigl[ f_{k} ( \xi _{k} ,\eta_{k} ) \mid \mathcal{F}_{a_{n},k-1} \bigr]\biggr\} \\ & \quad \leq \frac{\lambda }{2} \mathop{\lim\sup}_{n\rightarrow \infty } \frac{1}{b _{n}} \sum_{k= a_{n} +1}^{a_{n} + b_{n}} \mathbb{E} \bigl[ f _{k}^{2} ( \xi_{k}, \eta_{k} ) e^{\alpha \vert f_{k} ( \xi_{k} , \eta_{k} ) \vert } \mid \mathcal{F} _{a_{n}, k -1} \bigr] \\ & \quad =\frac{\lambda }{2}M(\alpha , \omega ) \quad \text{a.s. }\omega \in A( \alpha ). \end{aligned}$$

Letting $\lambda \searrow 0^{+} $ in Eq. (2.17), we get

2.18 $$ \mathop{\lim\sup}_{n\rightarrow \infty } \frac{1}{b_{n}} \sum _{k= a_{n} +1}^{a_{n} + b_{n}} \bigl\{ f_{k} ( \xi_{k}, \eta_{k} ) - \mathbb{E} \bigl[ f_{k} ( \xi_{k} ,\eta_{k} ) \mid \mathcal{F}_{a_{n},k-1} \bigr] \bigr\} \leq 0 \quad \text{a.s. }\omega \in A(\alpha ). $$

Taking $- \alpha < \lambda \leq 0$, similarly, we have

$$\begin{aligned}& \lim \inf_{n\rightarrow \infty } \frac{1}{b_{n}} \sum _{k= a_{n} +1} ^{a_{n} + b_{n}} \bigl\{ f_{k} ( \xi_{k}, \eta_{k} ) - \mathbb{E} \bigl[ f_{k} ( \xi_{k}, \eta_{k} ) \mid \mathcal{F}_{a_{n},k-1} \bigr] \bigr\} \\ & \quad \geq \frac{\lambda }{2} M(\alpha , \omega ) \quad \text{a.s. }\omega \in A( \alpha ). \end{aligned}$$

Putting $\lambda \nearrow 0^{-}$, we have

2.19 $$ \lim \inf_{n\rightarrow \infty } \frac{1}{b_{n}} \sum _{k= a_{n} +1} ^{a_{n} + b_{n}} \bigl\{ f_{k} ( \xi_{k}, \eta_{k} ) - \mathbb{E} \bigl[ f_{k} ( \xi_{k}, \eta_{k} ) \mid \mathcal{F}_{a_{n},k-1} \bigr] \bigr\} \geq 0 \quad \text{a.s. } \omega \in A(\alpha ). $$

From Eqs. (2.18) and (2.19), we obtain

$$ \lim_{n\rightarrow \infty } \frac{1}{b_{n}} \sum _{k= a_{n} +1}^{a_{n} + b_{n}} \bigl\{ f_{k} ( \xi_{k} ,\eta_{k} ) - \mathbb{E} \bigl[ f_{k} ( \xi_{k}, \eta_{k} ) \mid \mathcal{F}_{a_{n},k-1} \bigr] \bigr\} =0\quad \text{a.s. }\omega \in A(\alpha ). $$

Thus we complete the proof of Lemma 1. □

### Lemma 2

*Assume that* $\zeta = ( \xi , \eta ) = \{ ( \xi_{k}, \eta_{k} ) \} _{k=0}^{\infty }$ *is an HTIMC defined as in Lemma *1. *Then*, *for every* $j< k$; $k \geq 1$,

2.20 $$ \mathbb{E} \bigl[ f_{k} ( \xi_{k}, \eta_{k} ) \mid \mathcal{F}_{j,k-1} \bigr] = \mathbb{E} \bigl[ f_{k} ( \xi_{k}, \eta_{k} ) \mid \eta_{k-1} \bigr] \quad \textit{a.s.} $$

### Proof

From definition of Hidden Markov chain, we have, for every $x_{i} \in \mathcal{X}$, $y_{j}\in \mathcal{Y}$, $m \leq n$; $n \geq 1$,
 Hence, we have
 □

According to Theorem 1 of Wang [5], it is easy to verify the following lemma.

### Lemma 3

*Suppose that* $\eta = ( \eta_{0} , \eta_{1},\ldots )$ *is a time inhomogeneous Markov chain which takes value in state space* $\mathcal{Y}$, *its starting distribution is*

2.21 $$ \bigl(q(\omega _{0} ), q(\omega _{1} ); \ldots ; q( \omega_{b})\bigr),\quad q(\omega_{i}) > 0, \omega_{i} \in \mathcal{Y}, $$

*and transition matrices are*

2.22 $$ \mathcal{Q} _{k} = \bigl( q _{k} ( \omega_{j } \mid \omega_{i} )\bigr), \quad q _{k} ( \omega_{j } \mid \omega_{i} ) > 0, \omega_{i} , \omega_{j} \in \mathcal{Y} , k \geq 1, $$

*where*

$$q _{k} ( \omega_{j} \mid \omega_{i} ) = \mathbb{P} ( \eta _{k} = \omega_{j} \mid \eta _{k-1} = \omega_{i} ), \quad k \geq 1. $$

*Assume that* $\Pi = ( q ( \omega_{i} , \omega_{j} ))$, $q ( \omega_{i} , \omega_{j} ) > 0$, $\omega_{i} , \omega_{j} \in \mathcal{Y}$ *is another transition matrix which satisfies the following condition*:

2.23 $$ \lim_{n\rightarrow \infty } \frac{1}{b_{n}} \sum _{k= a_{n} +1}^{a_{n} + b_{n}} \bigl\vert q_{k} ( \omega_{i}, \omega_{j} ) - q ( \omega_{i}, \omega_{j} ) \bigr\vert =0 \quad \forall \omega_{i}, \omega_{j} \in \mathcal{Y}. $$

*Then*, *for each* $\omega_{s} \in \mathcal{Y}$,

2.24 $$ \lim_{n\rightarrow \infty } \frac{1}{b_{n}} \sum _{k= a_{n} +1}^{a_{n} + b_{n}} 1_{ \{ \omega_{s} \} } ( \eta_{k-1} ) = \pi_{s} \quad \textit{a.s.}, $$

*where* $( \pi_{0}, \pi_{1},\pi_{2},\ldots, \pi_{b} )$ *is the stationary distribution determined by *Π.

## Main results

### Theorem 1

*Let* $\zeta = ( \xi , \eta ) = \{ ( \xi_{k}, \eta_{k} ) \} _{k=0}^{\infty }$ *be an HTIMC which takes values in* $\mathcal{X} \times \mathcal{Y}$, $f ( x , y )$ *be a function on* ${\mathcal{X} \times \mathcal{Y}}$.

*Let* $\Pi = ( q ( \omega_{i} , \omega_{j} ))$, $q ( \omega_{i} , \omega_{j} ) > 0$, $\omega_{i} , \omega_{j} \in \mathcal{Y}$ *be another transition matrix and* $p ( \theta_{i} \mid \omega_{j} ), ( \theta_{i}, \omega_{j} ) \in \mathcal{X} \times \mathcal{Y}$ *be conditional probabilities which satisfy*

3.1 $$\begin{aligned}& \lim_{n\rightarrow \infty } \frac{1}{b_{n}} \sum _{k= a_{n} +1}^{a_{n} + b_{n}} \bigl\vert q_{k} ( \omega_{i}, \omega_{j} ) - q ( \omega_{i}, \omega_{j} ) \bigr\vert =0 \quad \forall \omega_{i}, \omega_{j} \in \mathcal{Y}, \end{aligned}$$

3.2 $$\begin{aligned}& \lim_{n\rightarrow \infty } \frac{1}{b_{n}} \sum _{k= a_{n} +1}^{a_{n} + b_{n}} \bigl\vert p_{k} ( \theta_{i }\mid \omega_{j} ) - p ( \theta_{i} \mid \omega_{j} ) \bigr\vert =0 \quad \forall ( \theta_{i}, \omega _{j} ) \in \mathcal{X}\times \mathcal{Y}. \end{aligned}$$

*If the transition matrix* Π *has a stationary distribution* $\pi = ( \pi_{0}, \pi_{1}, \pi_{2},\ldots, \pi_{b} )$, *then*

3.3 $$ \lim_{n\rightarrow \infty } \frac{1}{b_{n}} \sum _{k= a_{n} +1}^{a_{n} + b_{n}} f ( \xi_{k} ,\eta_{k} ) = \sum_{\theta_{i} \in \mathcal{X}} \sum_{\omega _{j} \in \mathcal{Y}} \sum_{\omega_{s} \in \mathcal{Y}} \pi_{s} f ( \theta_{i} ,\omega_{j} ) q ( \omega_{s}, \omega_{j} ) p( \theta_{i} \mid \omega_{s} ) \quad \textit{a.s.} $$

### Proof

Since $f ( x, y )$ is bounded, we have by Lemmas 1 and 2 that

3.4 $$ \lim_{n\rightarrow \infty } \frac{1}{b_{n}} \sum _{k= a_{n} +1}^{a_{n} + b_{n}} \bigl\{ f ( \xi_{k}, \eta_{k} ) - \mathbb{E} \bigl[ f ( \xi_{k}, \eta_{k} ) \mid \eta_{k-1} \bigr]\bigr\} =0 \quad \text{a.s.} $$

Observe that

$$ \mathbb{E} \bigl[ f ( \xi_{k}, \eta_{k} ) \mid \eta_{k-1} \bigr] = \sum_{\theta _{i} \in \mathcal{X}} \sum _{\omega _{j} \in \mathcal{Y}} f ( \theta_{i}, \omega _{j} ) q_{k} ( \eta_{k-1}, \omega_{j} ) p _{k} ( \theta_{i} \mid \eta_{k-1} ) . $$

We have that, by Eq. (3.4),

$$\begin{aligned}& \lim \sup_{n\rightarrow \infty } \Biggl\vert \frac{1}{b_{n}} \sum _{k= a_{n} +1} ^{a_{n} + b_{n}} f ( \xi_{k} ,\eta_{k} ) - \sum_{\theta_{i} \in \mathcal{X}} \sum_{\omega _{j} \in \mathcal{Y}} \sum_{\omega_{s} \in \mathcal{Y}} \pi_{s} f ( \theta_{i}, \omega_{j} ) q ( \omega_{s}, \omega_{j} ) p ( \theta_{i} \mid \omega_{s} ) \Biggr\vert \\& \quad \leq \lim \sup_{n\rightarrow \infty } \Biggl\vert \frac{1}{b_{n}} \sum_{k= a_{n} +1}^{a_{n} + b_{n}} \mathbb{E} \bigl[ f ( \xi_{k} ,\eta_{k} ) \mid \eta_{k-1} \bigr] - \sum _{\theta_{i} \in \mathcal{X}} \sum_{\omega _{j} \in \mathcal{Y}} \sum _{\omega_{s} \in \mathcal{Y}} \pi_{s} f ( \theta_{i}, \omega_{j} ) q ( \omega_{s}, \omega_{j} ) p ( \theta_{i} \mid \omega_{s} ) \Biggr\vert \\& \quad =\lim \sup_{n\rightarrow \infty } \Biggl\vert \frac{1}{b_{n}} \sum _{k= a_{n} +1}^{a_{n} + b_{n}} \sum_{\theta _{i} \in \mathcal{X}} \sum_{\omega_{j} \in \mathcal{Y}} f ( \theta_{i} ,\omega _{j} ) q_{k} ( \eta_{k-1}, \omega_{j} ) p _{k} ( \theta_{i} \mid \eta_{k-1} ) \\& \quad\quad{} - \sum_{\theta_{i} \in \mathcal{X}} \sum _{\omega_{j} \in \mathcal{Y}} \sum_{\omega_{s} \in \mathcal{Y}} \pi_{s} f ( \theta_{i},\omega_{j} ) q ( \omega_{s}, \omega_{j} ) p ( \theta_{i} \mid \omega_{s} ) \Biggr\vert \\& \quad =\lim \sup_{n\rightarrow \infty } \Biggl\vert \frac{1}{b_{n}} \sum _{k= a_{n} +1}^{a_{n} + b_{n}} \sum_{\theta_{i} \in \mathcal{X}} \sum_{\omega_{j} \in \mathcal{Y}} \sum_{\omega_{s} \in \mathcal{Y}} 1_{ \{ \omega_{s} \} } ( \eta_{k-1} )\pi_{s} f ( \theta_{i},\omega_{j} ) q_{k} ( \omega_{s}, \omega_{j} ) p_{k} ( \theta_{i} \mid \omega_{s} ) \\& \quad\quad{} - \sum_{\theta_{i} \in \mathcal{X}} \sum _{\omega_{j} \in \mathcal{Y}} \sum_{\omega_{s} \in \mathcal{Y}} \pi_{s} f ( \theta_{i},\omega_{j} ) q ( \omega_{s}, \omega_{j} ) p ( \theta_{i} \mid \omega_{s} ) \Biggr\vert \\& \quad =\lim \sup_{n\rightarrow \infty } \Biggl\vert \frac{1}{b_{n}} \sum _{k= a_{n} +1}^{a_{n} + b_{n}} \sum_{\theta_{i} \in \mathcal{X}} \sum_{\omega_{j} \in \mathcal{Y}} \sum_{\omega_{s} \in \mathcal{Y}} 1_{ \{ \omega_{s} \} } ( \eta_{k-1} ) f ( \theta_{i},\omega_{j} ) \\& \quad\quad{}\times \bigl[\bigl(q_{k} ( \omega_{s}, \omega_{j} ) - q ( \omega_{s}, \omega_{j} ) \bigr)p_{k} ( \theta _{i} \mid \omega_{s} ) \\& \quad\quad{} + q ( \omega_{s}, \omega_{j} ) \bigl( p _{k} ( \theta_{i} \mid \omega_{s} ) - p ( \theta _{i} \mid \omega_{s} ) \bigr) + p ( \theta_{i} \mid \omega_{s} ) q ( \omega_{s}, \omega_{j} ) \bigr] \\& \quad\quad{} - \sum_{\theta_{i} \in \mathcal{X}} \sum _{\omega_{j} \in \mathcal{Y}} \sum_{\omega_{s} \in \mathcal{Y}} \pi_{s} f ( \theta_{i},\omega_{j} ) q ( \omega_{s}, \omega_{j} ) p ( \theta_{i} \mid \omega_{s} ) \Biggr\vert \\& \quad \leq \lim \sup_{n\rightarrow \infty } \sum_{\theta_{i} \in \mathcal{X}} \sum_{\omega_{j} \in \mathcal{Y}} \sum_{\omega_{s} \in \mathcal{Y}} \sup_{\theta_{i} \in \mathcal{X}, \omega_{j} \in \mathcal{Y}} \bigl\vert f ( \theta_{i},\omega_{j} ) \bigr\vert \Biggl\{ \Biggl|\frac{1}{b_{n}} \sum _{k= a_{n} +1}^{a_{n} + b_{n}} \bigl\vert q_{k} ( \omega_{s}, \omega_{j} ) - q ( \omega_{s}, \omega_{j} ) \bigr\vert \\& \quad\quad{} + \frac{1}{b_{n}} \sum_{k= a_{n}}^{a_{n} + b_{n}} \bigl\vert p_{k} ( \theta_{i} \mid \omega_{s} ) - p ( \theta_{i} \mid \omega _{s} ) \bigr\vert \Biggr|\Biggr\} \\& \quad =0. \end{aligned}$$

Therefore Eq. (3.3) holds. □

### Corollary 1

*Under the conditions of Theorem *1, *we have for each* $\theta_{i '} \in \mathcal{X}$, $\omega_{j '}, \omega_{s} \in \mathcal{Y}$,

3.5 $$ \lim_{n\rightarrow \infty } \frac{1}{b_{n}} \mathcal{S}_{a_{n}, b_{n}} ( \theta_{i '}, \omega_{j '} )= \sum_{\omega_{s} \in \mathcal{Y}} \pi _{s} q ( \omega_{s}, \omega_{j '} ) p ( \theta_{i '} \mid \omega_{s} ) \quad \textit{a.s.} $$

### Proof

Put $f ( x , y ) = 1_{ \{ \theta_{i '} \} } ( x ) 1_{ \{ \omega_{j '} \} } ( y )$, $( \theta_{i '}, \omega_{j '} ) \in \mathcal{X} \times \mathcal{Y}$ in Theorem 1. Then

$$\begin{aligned}& \lim_{n\rightarrow \infty } \frac{1}{b_{n}} \sum _{k= a_{n} +1}^{a_{n} + b_{n}} f ( \xi_{k},\eta_{k} ) - \sum_{\theta_{i} \in \mathcal{X}} \sum_{\omega_{j} \in \mathcal{Y}} \sum_{\omega_{s} \in \mathcal{Y}} \pi_{s} f ( \theta_{i},\omega_{j} ) q ( \omega_{s}, \omega_{j} ) p ( \theta_{i} \mid \omega_{s} ) \\& \quad = \lim_{n\rightarrow \infty } \frac{1}{b_{n}} \sum _{k= a_{n} +1}^{a _{n} + b_{n}} 1_{ \{ \theta_{i '} \} } ( \xi_{k} ) 1_{ \{ \omega_{j '} \} } ( \eta_{k} ) \\& \quad\quad{} - \sum_{\theta_{i} \in \mathcal{X}} \sum_{\omega_{j} \in \mathcal{Y}} \sum_{\omega_{s} \in \mathcal{Y}} 1_{ \{ \theta_{i '} \} } ( \theta_{i} ) 1_{ \{ \omega_{j '} \} } ( \omega_{j} ) \pi_{s} q ( \omega_{s}, \omega_{j} ) p ( \theta_{i} \mid \omega_{s} ) \\& \quad = \lim_{n\rightarrow \infty } \frac{1}{b_{n}} \mathcal{S}_{a_{n}, b _{n}} ( \theta_{i '}, \omega_{j '} )- \sum _{\omega_{s} \in \mathcal{Y}} \pi_{s} q ( \omega_{s}, \omega_{j '} ) p ( \theta_{i '} \mid \omega_{s} ) =0 \quad \text{a.s.} \end{aligned}$$

 □

### Corollary 2

*Under the assumptions of Theorem *1, *we have*, *for each* $\theta_{i^{\prime\prime}} \in \mathcal{X}$, $\omega_{s} \in \mathcal{Y}$,

3.6 $$ \lim_{n\rightarrow \infty } \frac{1}{b_{n}} \mathcal{T}_{a_{n}, b_{n}} ( \theta_{i^{\prime\prime}} )= \sum_{\omega_{s} \in \mathcal{Y}} \pi_{s} p ( \theta_{i^{\prime\prime}} \mid \omega_{s} ) \quad \textit{a.s.} $$

### Proof

Put $f ( x , y ) = 1_{ \{ \theta_{i^{\prime\prime}} \} } ( x ) $, $( x , y ) \in \mathcal{X} \times \mathcal{Y} $ in Theorem 1. Then

$$\begin{aligned}& \lim_{n\rightarrow \infty } \frac{1}{b_{n}} \sum _{k= a_{n} +1}^{a_{n} + b_{n}} f ( \xi_{k},\eta_{k} ) - \sum_{\theta_{i} \in \mathcal{X}} \sum_{\omega_{j} \in \mathcal{Y}} \sum_{\omega_{s} \in \mathcal{Y}} \pi_{s} f ( \theta_{i},\omega_{j} ) q ( \omega_{s}, \omega_{j} ) p ( \theta_{i} \mid \omega_{s} ) \\& \quad = \lim_{n\rightarrow \infty } \frac{1}{b_{n}} \sum _{k= a_{n} +1}^{a _{n} + b_{n}} 1_{ \{ \theta_{i^{\prime\prime}} \} } ( \xi _{k} ) - \sum_{\theta_{i} \in \mathcal{X}} \sum_{\omega_{j} \in \mathcal{Y}} \sum_{\omega_{s} \in \mathcal{Y}} 1_{ \{ \theta_{i^{\prime\prime}} \} } ( \theta_{i} ) \pi_{s} q ( \omega_{s}, \omega_{j} ) p ( \theta_{i} \mid \omega _{s} ) \\& \quad =\lim_{n\rightarrow \infty } \frac{1}{b_{n}} \mathcal{T}_{a_{n}, b _{n}} ( \theta_{i^{\prime\prime}} ) - \sum_{\omega_{s} \in \mathcal{Y}} \pi_{s} p ( \theta_{i^{\prime\prime}} \mid \omega_{s} ) =0 \quad \text{a.s.} \end{aligned}$$

 □
